# Editorial: The evolution, characterization, and role of human endogenous retroviruses in health and diseases

**DOI:** 10.3389/fcimb.2024.1449864

**Published:** 2024-07-08

**Authors:** Lei Jia, Yanmei Song, Mingyue Chen, Richard Y. Zhao, Lin Li

**Affiliations:** ^1^ State Key Laboratory of Pathogen and Biosecurity, Academy of Military Medical Sciences, Beijing, China; ^2^ Department of Microbiological Laboratory Technology, School of Public Health, Cheeloo College of Medicine, Shandong University, Key Laboratory for the Prevention and Control of Emerging Infectious Diseases and Biosafety, Jinan, Shandong, China; ^3^ National 111 Center for Cellular Regulation and Molecular Pharmaceutics, Key Laboratory of Fermentation Engineering, Hubei University of Technology, Wuhan, China; ^4^ School of Medicine, University of Maryland, Baltimore, MD, United States

**Keywords:** endogenous retroviruses, evolution, regulatory role, health and diseases, molecular mechanism

Retroviruses are capable of infecting a wide range of vertebrates, including fish, amphibians, reptiles, birds, and mammals (Johnson, 2019). Usually, retroviruses infect somatic cells and upon germ cells are infected they may be transmitted to offspring. Over hundreds of millions of years, vertebrate genomes have accumulated thousands of sequence loci for retroviruses due to germ cell infection by exogenous retroviral during evolution, which are currently known as endogenous retroviruses (ERVs) (Stoye, 2001; Jern and Coffin, 2008). Among them, human endogenous retroviruses (HERVs) have accounted for up to 8%-10% of the host genome (Grandi and Tramontano, 2018). The typical genome structure of ERVs mainly consists of the flanking long terminal repeat sequences (LTRs) at both ends and four open reading frames in the middle, *gag*, *pro*, *pol*, and *env*. Due to the accumulation of insertions, deletions, mutations, and intrastrand recombination, most ERV elements lack complete open reading frames currently (Jia et al., 2016; Jia and Li, 2018). And approximately 85-90% of ERV elements are solo LTRs resulting from recombination between the two flanking LTRs (Jia et al., 2022; Wang et al., 2024). HERVs have attracted increasing attention in recent years, especially since the Human Genome Project (Lander et al., 2001; Liu et al., 2022). A great deal of research have been devoted to their characterization, evolution, and biological functions. Recent evidence suggests that HERVs, which are distributed throughout the human genome, are a rich source of regulatory elements in the human genome (Chen et al., 2022). HERVs, as a type of transposable elements (TE), reshaped the host genome and can provide favorable conditions for regulating the expression of neighboring genes (Chen et al., 2022; Liu et al., 2022).

In this context, we launched this Research Topic and aimed to shed light on both the evolution and role of HERVs in human health and diseases (Vargiu et al., 2016; Costa and Vale, 2023). Da Silva et al. (Beyond pathogens: the intriguing genetic legacy of endogenous retroviruses in host physiology) reviewed the important roles played by ERVs in the regulation of host functional homeostasis, from early life embryogenesis and the coordinated activities of pluripotent genetic regulatory networks to the regulation of cellular senescence. Two HERV-W family genes, encoding the env glycoproteins syncytin-1 and syncytin-2, have been implicated in intercellular fusion and placentation (Denner, 2016). They function in the fusion of syncytiotrophoblast and chorionic trophoblast and play an important role in fetogenesis by protecting the fetus from maternal rejection (Bolze et al., 2017). Notably, another Env protein, Suppressyn, encoded by HERV-Fb1, has been recognized as a Syncytin-1-specific inhibitor. It can act as a regulatory mediator of syncytization and prevent abnormalities in the placenta. HERV not only affects embryogenesis but also plays a role in innate immune regulation. Grow, E.J. et al. reported that overexpression of Rec protein encoded by ERV-K in human pluripotent cells increased the innate antiviral response of the organism and inhibited exogenous viral infections (Grow et al., 2015). Chiappinelli et al. demonstrated in another example that ERV is involved in innate immunity through multiple mechanisms, that bi-directional transcription of ERV activates the triggering of the type I interferon response and the apoptotic double-stranded RNA sensing pathway (Chiappinelli et al., 2015).

Besides physiological effects, some studies have found that abnormal ERV activation is associated with cancer, autoimmune diseases, and neurodegenerative diseases (Stoye, 2012; Bhardwaj and Coffin, 2014; Küry et al., 2018). For example, HML-2 is the most biologically active subset of the HERV-K family, and the expression of its members is associated with many types of cancer. Further understanding of the characterization and roles of ERVs will provide us with new insights. Cherkasova et al. (Mechanistic regulation of HERV activation in tumors and implications for translational research in oncology) reviewed the role of ERVs in regulating immune activity in the tumor microenvironment. Wen et al. (Endogenous retrovirus group FRD member 1 is a potential biomarker for prognosis and immunotherapy for kidney renal clear cell carcinoma) performed a comprehensive analysis of clinical data from public databases and the results showed that ERV FRD-1 is lowly expressed in kidney renal clear cell carcinoma (KIRC) tissues compared to normal tissues. Interestingly, patients with elevated ERV FRD-1 expression had a better prognosis. To better understand the role of ERV FRD-1 in KIRC, its biological functions and signaling pathways were further analyzed. ERV FRD-1 was found to be closely associated with the appearance of immune markers, which may achieve anti-KIRC effects by activating the immune system. In addition, an exciting finding was that Suppressyn and Syncytin-2, previously known during embryogenesis, now appear to play important roles in acute myeloid leukemia (AML). In addition, Shen et al. (Endogenous retroviruses Suppressyn and Syncytin-2 as innovative prognostic biomarkers in Acute Myeloid Leukemia) reported that HERV was differentially expressed in control and Acute Myeloid Leukemia (AML) samples and that this difference correlated with the clinical characteristics of the patients. Suppressyn and Syncytin-2 were involved in enhancing the immune phenotype of AML and also had a significant effect on immune cell infiltration. This suggests that ERV may be involved in immune response and immunomodulation in AML. In conclusion, ERV is an important molecular marker for cancer immunotherapy, providing information on immune response and prognosis of the disease, and may also be used for vaccine development.

ERVs have an important influence on the physiological regulation of the organism as well as in the development of diseases. All of the above publications outline the symbiotic and conflicting mechanisms between endogenous retroviruses and humans. It is worth noting that although there have been studies showing the mechanisms of ERV activation in specific cancers and the association with cancer immuno-therapy, most of these are based on animal models and mining analysis of public databases. Our understanding of the mechanism of action of these ERV elements in health and disease-related pathways is far from adequate. Dysregulation of ERV expression in the organism does not appear to be random, with some expression being upregulated and some being downregulated in specific situations. Specific ERV family loci are turned on or off only when necessary to determine the fate of cells and tissues during a particular ontogeny. This also poses new challenges: where is the balance between ERV in physiological regulation and disease development? Further research is needed to elucidate the exact molecular mechanisms and pathways of ERVs in cancer and other diseases and to apply this knowledge to the development of new therapeutic approaches.

## Author contributions

LJ: Writing – original draft, Writing – review & editing, Conceptualization. YS: Writing – review & editing, Writing – original draft. MC: Writing – review & editing. RZ: Writing – review & editing. LL: Conceptualization, Writing – review & editing, Writing – original draft.
